# Intervertebral disc degeneration therapies in human

**DOI:** 10.1186/1471-2474-16-S1-S19

**Published:** 2015-12-01

**Authors:** Stephen M Richardson

**Affiliations:** 1 Centre for Tissue Injury and Repair, Institute of Inflammation and Repair, Faculty of Medical and Human Sciences, The University of Manchester, Oxford Rd, Manchester M13 9PL, UK

## 

Low back pain (LBP) is one of the most common musculoskeletal disorders, with an estimated 84% of the population experiencing LBP at some point in their lifetime. As with most musculoskeletal disorders, the prevalence of LBP increases with age, suggesting incidences of LBP are likely to increase in the future due to a global aging population, changes in lifestyle and occupational stresses. Although the causes of LBP are multifactorial, increasing evidence implicates intervertebral disc (IVD) degeneration as a major contributor, with loss of IVD integrity leading to the destabilization of the spinal motion segment, resulting in pain and disability.

The IVD is a complex structure that allows movement between adjacent vertebrae and sustains the load applied through the spine. It consists of the peripheral annulus fibrosus (AF), a ligamentous lamellar structure composed predominantly of type I collagen fibres, and the central nucleus pulposus (NP), a highly hydrated structure, composed of the proteoglycan aggrecan, interspersed with type II collagen fibres. Only 1% of the IVD volume is occupied by its constituent cells, but they assume a key role, as they maintain IVD homeostasis. In degeneration there is an alteration in NP cell biology leading to diminished cell numbers and altered cell function resulting in an imbalance between matrix synthesis and degradation, particularly within the NP.

Current medical treatments for IVD degeneration rely on conservative therapies (e.g. pain relief, exercise therapy) and, when these fail, surgery. Surgical treatments such as spinal fusion and disc replacement have shown satisfactory results in alleviating pain, but are not devoid of complications and long-term clinical outcomes still remain poor. Thus, there is an urgent need for alternative therapies focussed on correcting the underlying pathogenesis and aberrant cell biology of IVD degeneration. As such many researchers, including ourselves, are focussing on the development of novel cell-based therapies. However, in order for these to be successful an appropriate cell source for implantation and tissue regeneration must be identified.

In this presentation we discussed the pathophysiology of IVD degeneration, efforts to elucidate the phenotype of human IVD cells and how this has allowed development of mesenchymal stem cell (MSC)-based therapies for IVD regeneration. In particular it focused on our efforts to identify the optimal MSC source and growth factor to direct differentiation and enhance tissue formation, as well as the influence microenvironment has on regeneration strategies.

